# *QuickStats*: Percentage* of Adults Aged ≥18 Years Who Always Use Sunscreen When Outside for >1 Hour on a Sunny Day,^†^ by Sex and Age Group — National Health Interview Survey, United States, 2020^§^

**DOI:** 10.15585/mmwr.mm7122a5

**Published:** 2022-06-03

**Figure:**
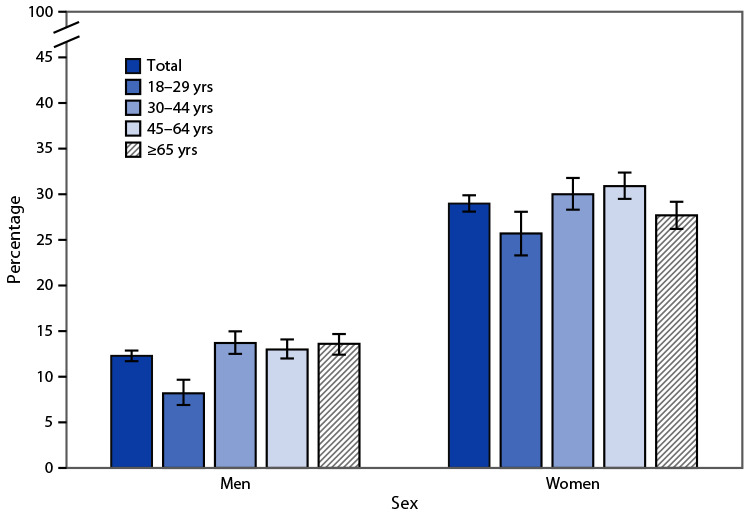
In 2020, 12.3% of men and 29.0% of women aged ≥18 years always used sunscreen when outside on a sunny day for >1 hour. The percentage of men who always used sunscreen was lowest among those aged 18–29 years (8.2%) and increased to 13.7% among those aged 30–44, 13.0% among those aged 45–64, and 13.6% among those aged ≥65 years. The percentage of women who always used sunscreen was lower among those aged 18–29 and ≥65 years (25.7% and 27.7%, respectively) compared with those aged 30–44 and 45–64 years (30.0% and 30.9%, respectively). For every age group, women were more likely than men to always use sunscreen.

**Source:** National Center for Health Statistics, National Health Interview Survey, 2020. https://www.cdc.gov/nchs/nhis.htm

